# A Huge High-Flow Aneurysmal Renal Arteriovenous Malformation Treated With Endovascular Transcatheter Embolization

**DOI:** 10.7759/cureus.65487

**Published:** 2024-07-27

**Authors:** Maciej Mach, Karol Maciejewski, Tomasz Ostrowski, Rafał Maciąg, Michał Sajdek, Oskar Gąsiorowski, Zbigniew Gałązka

**Affiliations:** 1 Department of General, Vascular, Endocrine and Transplant Surgery, Medical University of Warsaw, Warsaw, POL; 2 2nd Department of Clinical Radiology, Medical University of Warsaw, Warsaw, POL

**Keywords:** renal intervention, vascular plug, arteriovenous fistula, endovascular embolization, renal arteriovenous anomaly

## Abstract

Renal arteriovenous anomalies are uncommon. They are characterized by an abnormal vascular connection that usually bypasses the capillary bed. Most are acquired arteriovenous fistulas (AVF) while the rest are congenital or idiopathic arteriovenous malformations (AVM). AVF are usually caused by renal interventions, trauma, or neoplastic processes. They can lead to hypertension, heart failure, hematuria, and renal insufficiency. A 69-year-old woman presented with arrhythmia, tachycardia, mild ankle edema, and increasing fatigue. Right kidney color Doppler ultrasound confirmed the presence of a huge AVM with a blood flow of 9 L/minute and a dilated, 35 mm in diameter, right renal vein. Two months later, an attempt to embolize the AVM failed as the Amplatzer™ Vascular Plug II (Abbott Laboratories, Chicago, Illinois, United States) migrated to the pulmonary circulation and was later removed. Complete embolization was achieved by implanting two Amplatzer Vascular Plug IIs, various embolization coils, histoacryl glue, and lipiodol. Control angiography revealed significant stenosis in the right subclavian artery endovascular access, which was managed with BeGraft (Bentley InnoMed GmbH, Hechingen, Germany) and Zilver (Cook Group Incorporated, Bloomington, Indiana, United States) stents. The patient was discharged on the third postoperative day, all her symptoms resolved, and she reported eventual recovery. Three months later, the patient was operated on due to a 40x58 mm pseudoaneurysm at the right femoral access site. Thus, renal AVMs should be included as a potential alternative diagnosis for various symptoms such as hematuria and hypertension resistant to medication. Endovascular embolization is a less-invasive, safer, and more effective option than open surgery but has a risk of complications. Success requires fully occluding the shunted vessel, preventing embolic material migration, and preserving normal arterial branches. It depends on selecting adequate techniques and embolic materials individually, based on etiology and precise vascular anatomy assessment.

## Introduction

Renal arteriovenous anomaly is a condition in which there is a pathological connection between the arterial and venous systems. They are relatively rare with an incidence of around 0.04% and can be divided into acquired and congenital types [1,2]. The congenital type consists of arteriovenous malformation (AVM), which according to Yakes AVM classification can be divided into the following types of lesions: type I, a direct arteriovenous fistula (AVF), type IIa, which is a typical nidus AVM; type IIb, nidus AVM with aneurysmal venous drainage, type IIIa with multiple in-flow arterioles shunting into an aneurysmal vein, type IIIb with multiple in-flow arterioles and multiple out-flow veins with aneurysmal vein, and type IV with multiple arteries/arterioles that form many micro-fistulae and infiltrate the tissue [3].

AVF is an acquired abnormality, most commonly associated with renal trauma, neoplastic process, or surgical intervention on the renal system. AVF are defined by a single direct communication between arterial and venous vessels lacking an intervening vascular nidus [4]. AVF and AVM present with a range of symptoms such as high-output congestive heart failure, refractory hypertension, hematuria, and abdominal pain [5]. Historically, renal AVF and AVM were managed surgically, typically involving the ligation of the arterial feeder, nephrectomy, or partial nephrectomy [6]. However, the primary approach to treatment favors endovascular methods, commonly involving coil embolization [7,8]. Unfortunately, these methods might lead to complications such as migration of embolic agents into the venous and pulmonary circulation, as well as extensive hemorrhage, formation of a pseudoaneurysm, and endovascular access stenosis [8]. We present a case of a woman with a huge, high-flow renal AVM treated with percutaneous embolization.

## Case presentation

A 69-year-old female with a history of arrhythmia, tachycardia episodes, and mild ankle edema was admitted to the cardiology department of a local hospital in July 2023. She complained of dyspnea and gradually increasing fatigue within the last eight months. Besides that, family history was unremarkable for reno-vascular disease and there was no history of previous renal interventions, trauma, or neoplastic processes. Imaging and laboratory tests revealed signs of heart failure, prompting further investigation. A chest radiograph demonstrated a significantly enlarged cardiac silhouette and pulmonary circulation congestion. A right kidney Doppler ultrasound showed a significant high-flow vascular malformation with blood flow through the right renal artery of approximately 8/9 L/minute and dilatation of the right renal vein to 35 mm. Echocardiography showed enlargement of all four heart chambers, especially the atria. The sizes of the right and left ventricles were 44 mm and 55 mm, respectively. Significant hypertrophy of the left ventricular muscle was also demonstrated. The ejection fraction was 55%.

**Figure 1:**
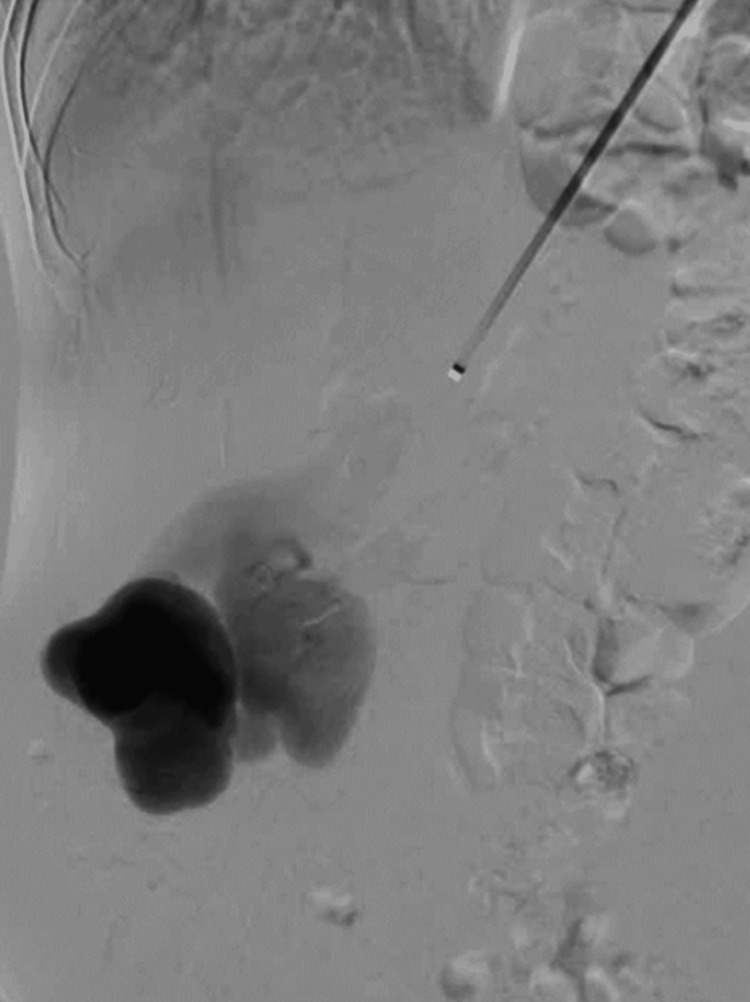
Large right renal arteriovenous malformation through a dilated and elongated renal artery with aneurysmal dilatation of the venous end.

In September 2023, an initial embolization attempt of the AVM at a vascular surgery department of a different hospital was unsuccessful. During the procedure, an Amplatzer™ Vascular Plug II (Abbott Laboratories, Chicago, Illinois, United States) device migrated toward the venous part of the AVM, necessitating its removal. Due to technical challenges, the patient was admitted to a reference center for further management. In October 2023, endovascular transcatheter embolization was conducted at the 2nd Department of Clinical Radiology at our institute. During the endovascular therapy, selective right kidney artery angiography through the right subclavian artery confirmed the presence of a high-flow vascular malformation accompanied by aneurysmal dilatations of the arterial part of the malformation and varicose dilatations of the outflow part (Figure 1).

**Figure 2:**
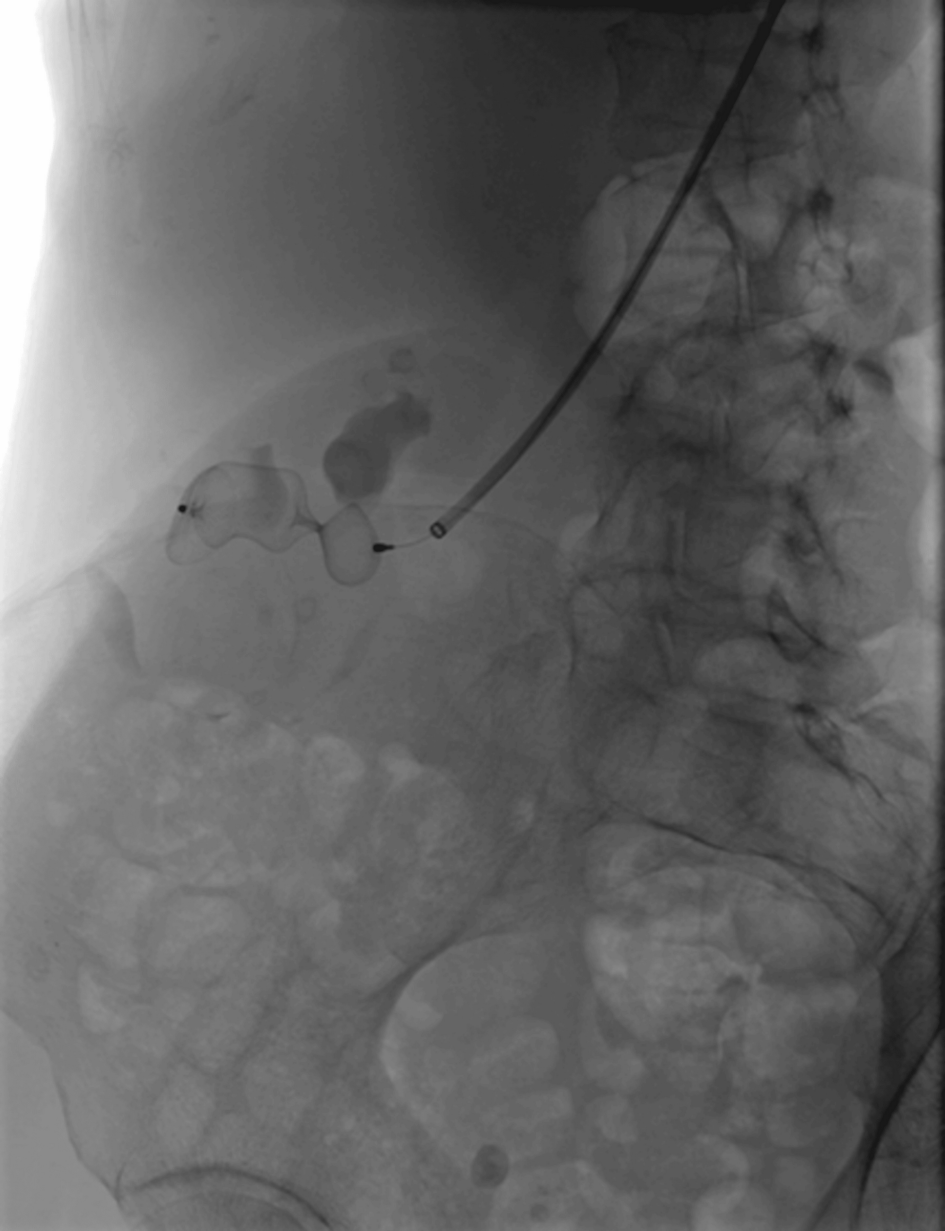
Placement of the first Amplatzer™ type II occluder into the right renal artery. A reduction in the blood flow through the arteriovenous malformation is demonstrated. Amplatzer™ type II occluder, Abbott Laboratories, Chicago, Illinois, United States

At the same time, after inserting a FLEXOR 8F Guiding Sheath (Cook Group Incorporated, Bloomington, Indiana, United States) into the distal section of the right renal artery trunk, a 22 mm Amplatzer Type II occluder (Abbott Laboratories) was implanted into its lumen. Follow-up angiography revealed a decrease in flow dynamics within the AVM (Figure 2).

**Figure 3:**
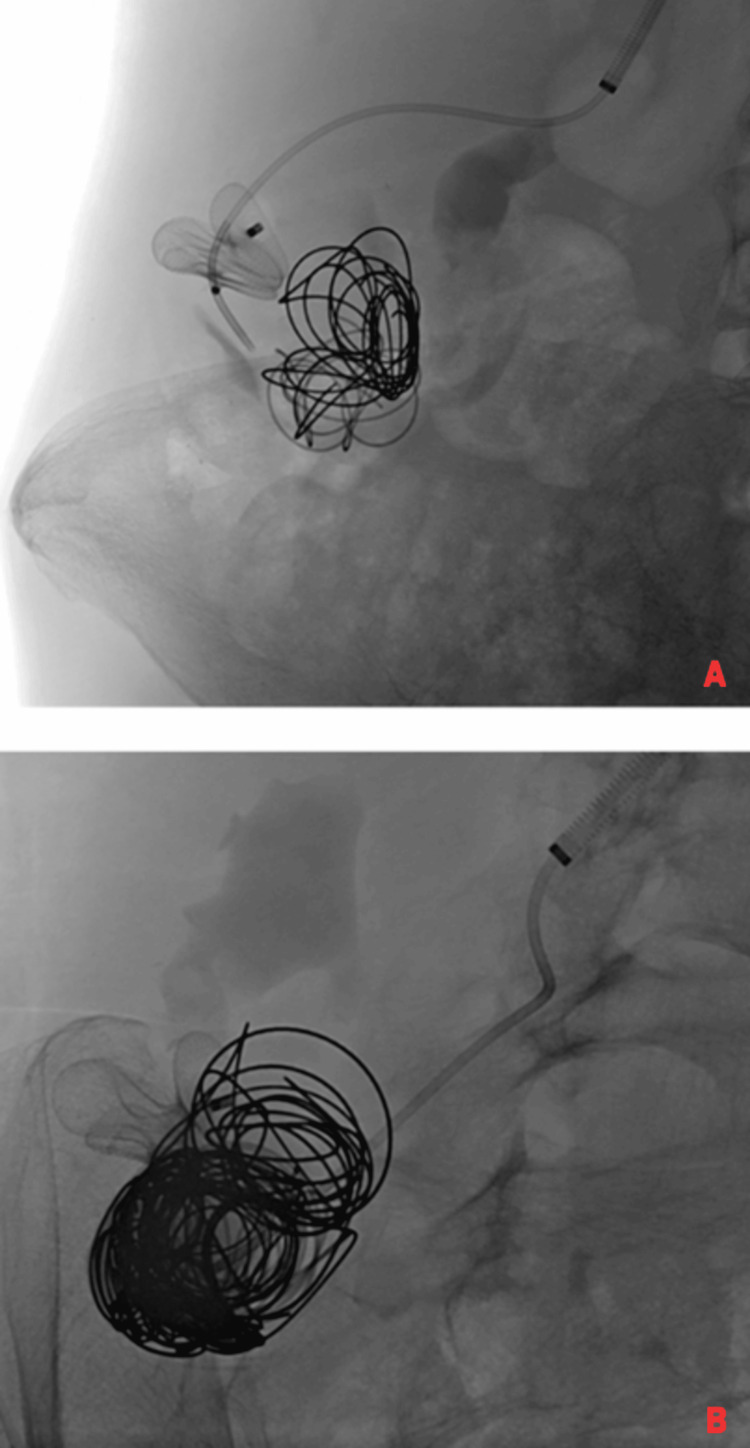
Distal migration of the previously implanted Amplatzer™ type II occluder. (A) Visible AMPLATZER type II occluder migration; (B) Embolization coils placed in the arteriovenous malformation. Amplatzer™ type II occluder, Abbott Laboratories, Chicago, Illinois, United States

In the next stage, a microcatheter was introduced into the lumen of the aneurysmal dilatation of the AVM distally to the implanted occluder, and embolization of the dilated part was performed using MReye® and Nester® embolization coils (Cook Group Incorporated), four detachable AZUR™ HydroCoil (Terumo Corporation, Shibuya City, Tokyo, Japan), and 28 Ruby Standard embolization coils (Penumbra, Inc., California, United States) (Figure 3).

**Figure 4:**
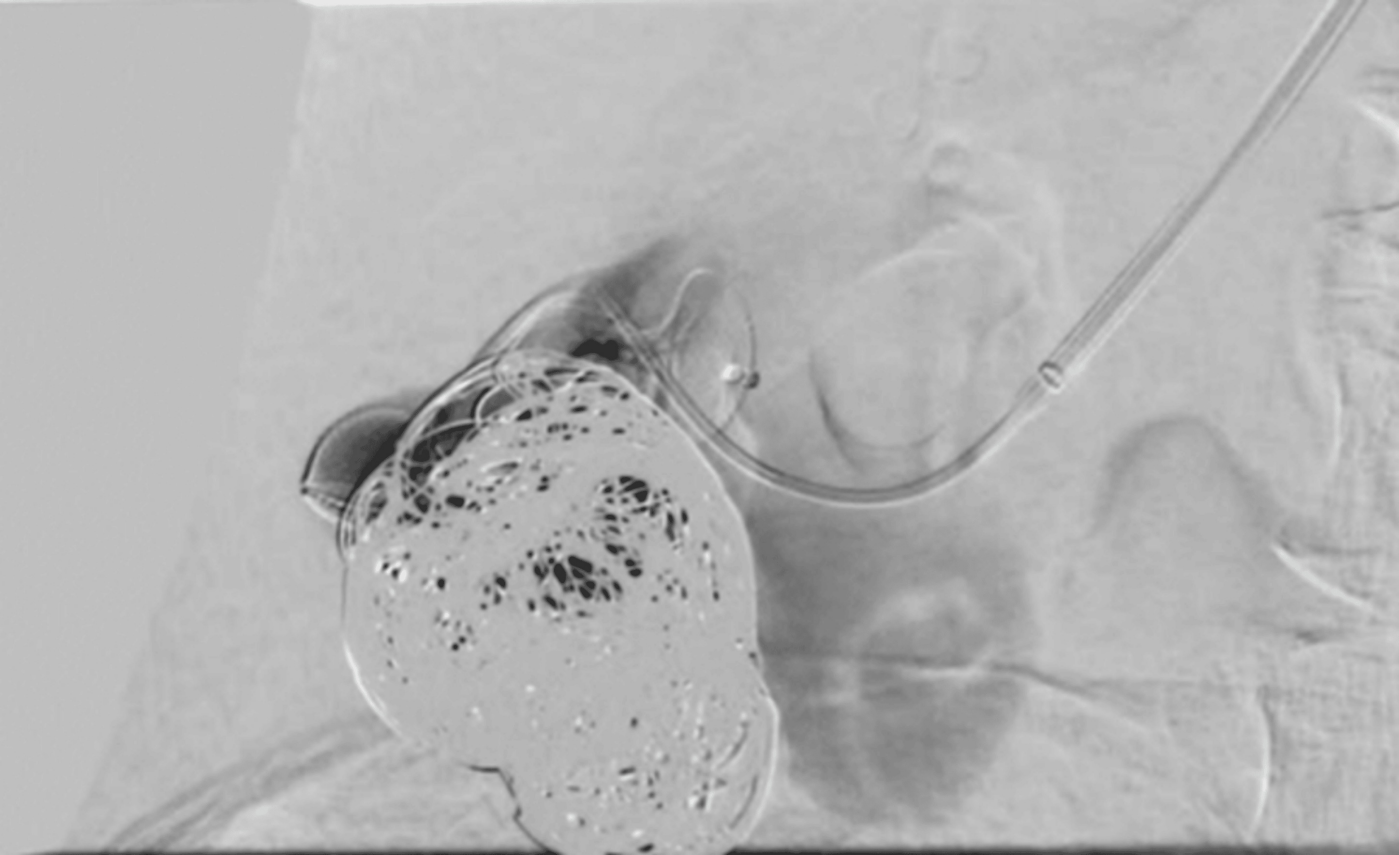
Partial closure of the arteriovenous malformation after implanting the second Amplatzer™ type II occluder. Amplatzer™ type II occluder, Abbott Laboratories, Chicago, Illinois, United States

Due to the displacement of the previously implanted occluder distally to the aneurysmal dilatation of the malformation, another Amplatzer Type II occluder with a diameter of 22 mm was implanted into the lumen of the distal section of the right renal artery trunk, without achieving complete occlusion of the AVM (Figure 4).

After inserting the PROGREAT® 2.7 microcatheter (Terumo Corporation) between the occluder modules, it was sealed using detachable Ruby Standard embolization coils, seven Packing Coils (Penumbra, Inc.) and a mixture of histoacryl glue and lipiodol at a concentration of 50%, achieving complete occlusion of the AVM.

**Figure 5:**
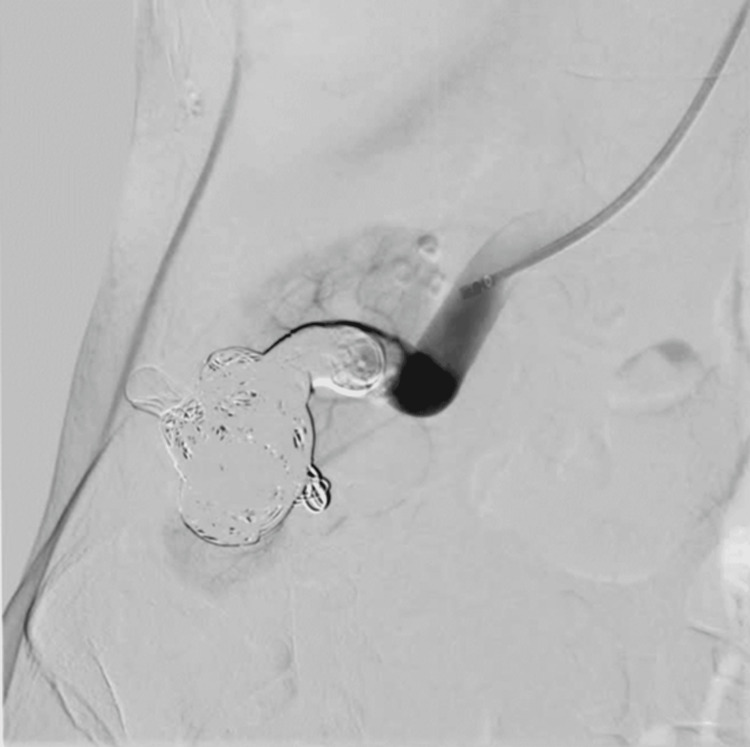
Post embolization angiography demonstrating normal right kidney nephrogram and a full occlusion of the arteriovenous malformation.

Follow-up angiography confirmed a normal nephrogram of the right kidney with complete exclusion of AVM (Figure 5).

**Figure 6:**
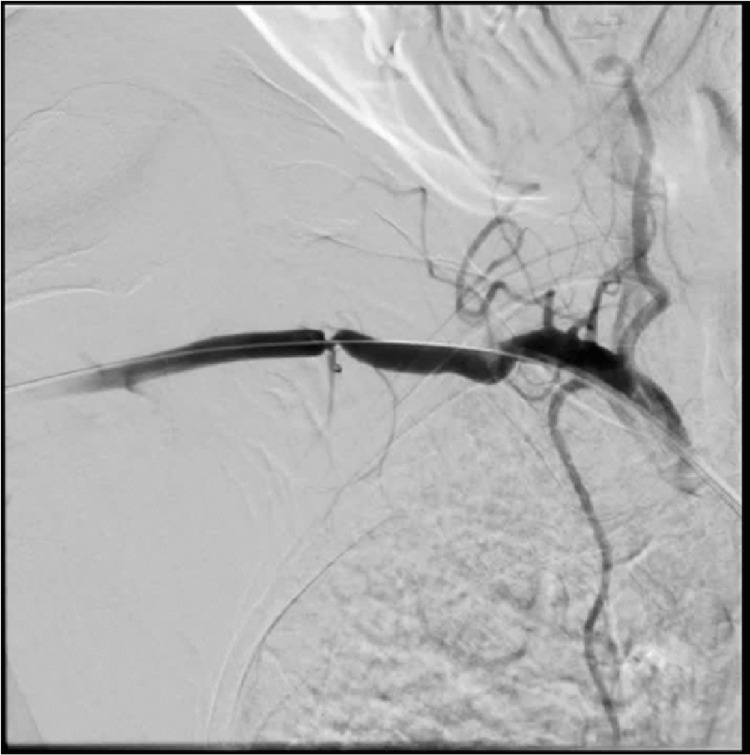
Right subclavian artery angiography demonstrating a significant stenosis of the arterial access site.

The site of endovascular access to the right subclavian artery was secured using two ProGlide/ProStyle™ occlusion systems (Abbott Laboratories). Angiography of the right subclavian artery performed through the right femoral artery access showed significant hemodynamic stenosis at the arterial access site (Figure 6).

**Figure 7:**
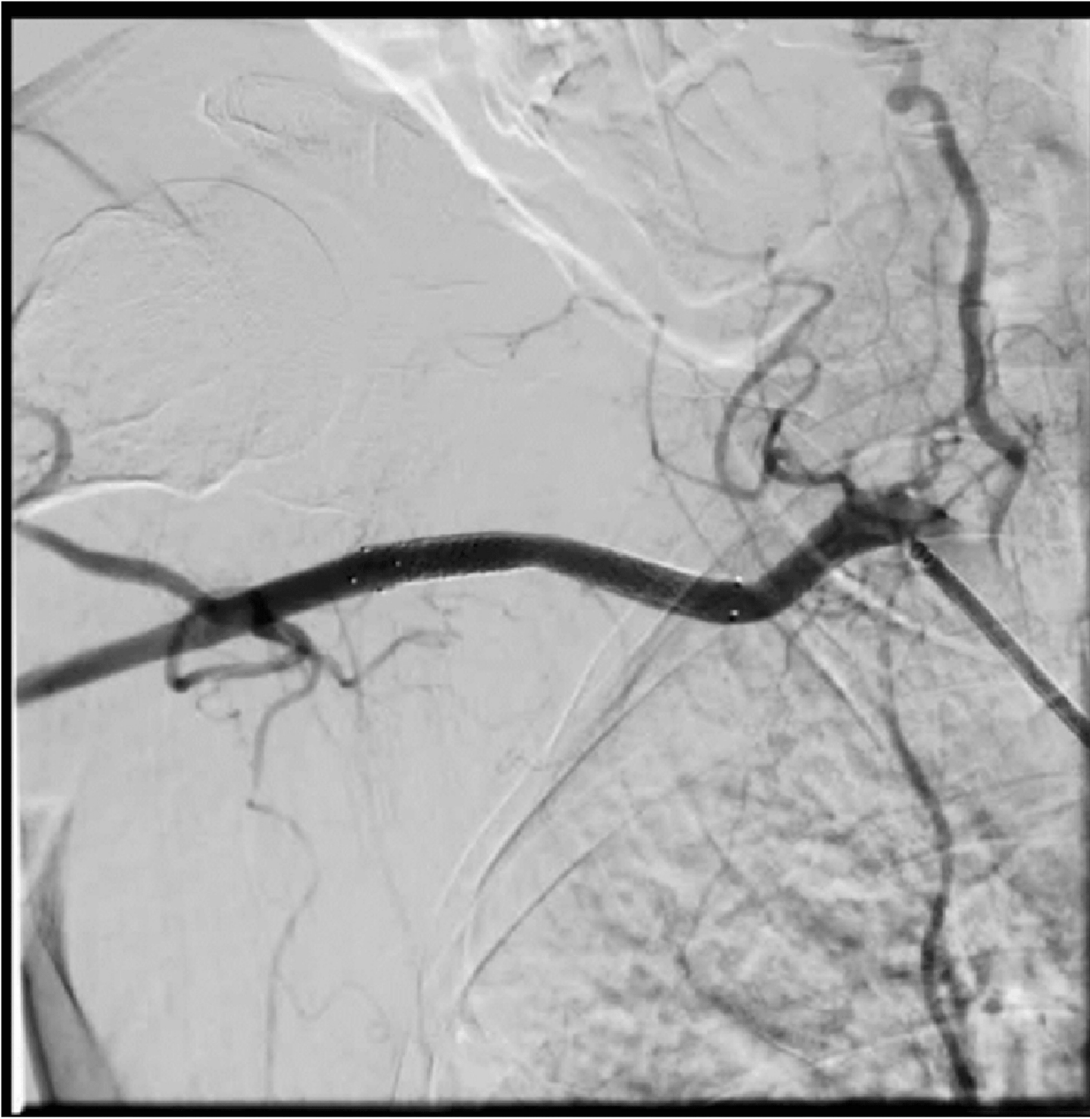
Successful implantation of covered BeGraft* and self-expanding Zilver** stents into the right subclavian artery. * BeGraft 7x57 (Bentley InnoMed GmbH, Hechingen, Germany); ** Zilver 8x60 mm (Cook Group Incorporated, Bloomington, Indiana, United States)

Therefore a percutaneous transcatheter angioplasty of the narrowed right subclavian artery was performed. A covered BeGraft 7x57 stent (Bentley InnoMed GmbH, Hechingen, Germany) was implanted into the subclavian trunk, supported by a self-expanding Zilver 8x60 mm stent (Cook Group Incorporated) (Figure 7).

**Figure 8:**
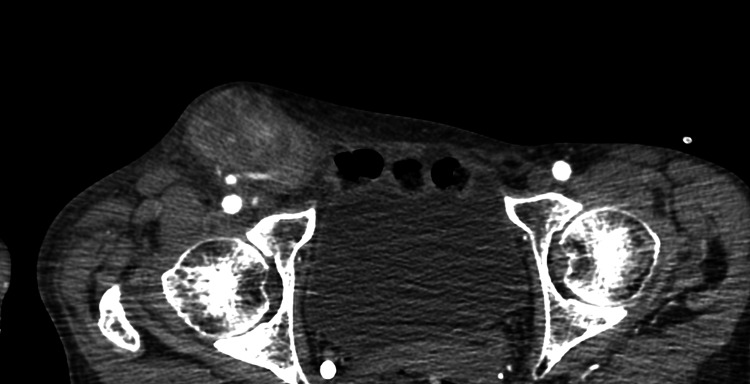
Postoperative pseudoaneurysm formation at the right femoral vascular access site.

The patient's hospitalization was uncomplicated. She was discharged home on the third postoperative day. On follow-up, the patient reported a reduction in symptoms and overall health improvement. The only reported complication was the development of a palpable 40x58 mm pseudoaneurysm at a right femoral access site, which caused pain and a deterioration in the quality of life (Figure 8).

**Figure 9:**
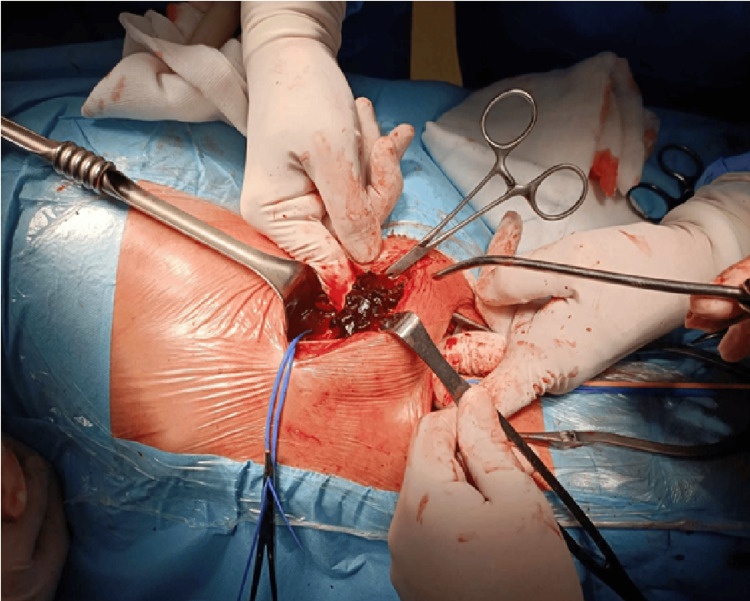
Intraoperative photo of the pseudoaneurysm surgical removal.

The pseudoaneurysm was surgically removed in December 2023 (Figure 9).

## Discussion

Etiology

The most common subtype of vascular anomalies is acquired AVF, which can be caused by renal trauma, tumor neovascularity, or inflammation affecting the vessel wall. The time span from the initial trauma to the diagnosis can range from a few weeks to years [9]. Since our patient did not experience any predisposing events, the congenital or idiopathic subtype is more likely. Nevertheless, the precise moment of malformation emergence in our patient is unclear, so the definitive cause cannot be identified. 

Clinical manifestation

The symptoms of AVF and AVM vary based on their location and size. The typical clinical manifestation of renal AVM is the presence of micro- or macrohematuria [10]. Other common symptoms include side pain, a slight increase in heart rate, elevated blood pressure, vertigo, overall fatigue, auscultatory phenomena in the renal arteries due to turbulent blood flow, and symptoms suggestive of congestive heart failure [10-12]. If not addressed, chronic alterations linked with AVM, involving deterioration of ventricular dilation and function, left ventricular hypertrophy, and pulmonary hypertension may lead to detrimental remodeling of the heart exacerbating myocardial dysfunction [13]. This case highlights the complexities involved in diagnosing renal AVM, particularly those presenting with high-output cardiac symptoms. Our patient's symptoms of arrhythmia, tachycardia, and fatigue were atypical for AVM, thus the patient was initially diagnosed in the cardiology department before identifying the renal etiology.

Diagnostics

Imaging studies are crucial for both diagnosing and treating vascular abnormalities; nevertheless, they can pose challenges. Tools used in diagnostics include color Doppler ultrasound (CDU), magnetic resonance imaging (MRI), computed tomography (CT), and angiography [14,15]. CT, along with ultrasound, is usually the first option, used to eliminate other potential conditions [16]. CDU is helpful in visualizing fast-flow arterio-venous shunts whereas MRI is especially useful in complex arteriovenous anomalies. Time-resolved four-dimensional CT angiography (4D CTA) helps to find the nidus and analyze the blood flow patterns of inflow and outflow vessels. Furthermore, different projections are important in evaluating access for embolotherapy [14].

CDU is usually the initial diagnostic test, due to its inexpensiveness, non-invasiveness and wide availability. In grayscale ultrasound, renal AVF might appear anechoic, resembling a renal cyst. Hence, CDU becomes crucial not just for identifying the vascular characteristics of the lesion but also for illustrating arterialized venous return. Furthermore, spectral analysis reveals elevated flow velocity, reduced arterial resistance, and arterial waveforms within the draining vein [17]. CDU is highly valuable for evaluating renal cystic lesions, aiding in distinguishing between simple and complicated cysts, identifying vascular issues, and depicting the vascularization of septae or solid components within cystic lesions [18]. Yet, diagnosing type III is challenging with ultrasonography, likely due to the intricate, minuscule, abnormal vascular networks that are hard to identify.

Despite the fact that angiography is significantly more invasive than ultrasonography and CT scanning, it may provide valuable diagnostic and treatment insights in suspicious cases.

Due to symptoms of arrhythmia, tachycardia, and mild ankle edema, our patient was initially admitted to the cardiology department. Imagining findings such as significantly enlarged cardiac silhouette and pulmonary circulation congestion in chest radiographs, enlargement of all four heart chambers, especially the atria, and significant hypertrophy of the left ventricular muscle, with an ejection fraction of 55% in the echocardiography suggested heart failure. A right kidney CDU revealed a substantial high-flow vascular malformation with blood flow through the right renal artery of around 8/9 L/minute and dilatation of the right renal vein to 35 mm. These CDU findings were pivotal in diagnosing renal AVM.

Treatment

Management of renal AVF ranges from monitoring for patients without symptoms to surgical procedures for symptomatic patients, offering both endovascular and open surgical options. Before choosing the treatment approach, factors such as the presence and intensity of ailments, the patient's age, blood pressure, existing medical conditions, renal function, and vascular anatomy along with the facility’s experience and available technology should be taken into account. The therapeutic goal for renal AVF is to preserve functional nephrons to the greatest extent possible, while also alleviating symptoms and correcting hemodynamic abnormalities [19]. Currently, the main treatment options consist of surgical procedures and interventional embolization. AVF discovered incidentally without any accompanying symptoms are treated with conservative management.

Open Surgery

Surgical treatment such as nephrectomy or ligation of the renal artery has numerous drawbacks, including its invasive nature, high chance of renal parenchymal and functional loss leading to increased rates of morbidity and mortality, potential complications such as substantial hemorrhage, and the requirement for extended hospital stays [20].

Endovascular Embolization

Endovascular embolization has become the primary treatment option for AVF cases, as it provides precise and effective closure of the vascular abnormality while preserving renal function to the maximum achievable degree. In comparison to open surgery, endovascular treatment is less limited by the risk of bleeding, vascular damage, and the scope of the procedure.

After consulting with interventional radiologists, considering the technical capabilities of our Department, the lesser invasiveness of the endovascular procedure, and the lesser risk of complications, we decided to perform percutaneous endovascular embolization.

Materials

Various embolization materials have been utilized, such as metallic coils, sclerosing liquid agents, particulate embolization materials, covered stents, and vascular plugs. The choice of embolic material is dependable on factors like the anatomical structure of blood vessels and hemodynamics, the specific type of vascular pathology, as well as desired technical and clinical outcomes [21]. Given the size of the AVM, the high blood flow through the AVM, and the failure of the previous embolization attempt in our patient, we decided to use a combination of Amplatzer Type II Vascular Plug, embolization coils, histoacryl glue, and lipiodol to ensure complete occlusion and to prevent recurrence.

Complications

While uncommon, complications such as the occlusion of nearby or intact proximal vessels are possible, leading to significant loss of renal parenchyma and embolization agent migration, resulting in a potential pulmonary embolism [22]. It is expected that less than 25% of parenchyma loss may occur during the treatment, usually caused by the administration of nephrotoxic iodinated contrast and reduction of blood supply to a section of the renal parenchyma [23,24]. However, this generally does not impact overall renal function. In high-flow AVF, embolization with coils presents technical challenges primarily due to the risk of embolic material migration into an already dilated inferior vena cava and potentially even into the pulmonary circulation [25]. Although rare, endovascular treatment carries a risk of iatrogenic pseudoaneurysms at the vascular access sites, as well as potential vascular stenosis [26]. Endovascular treatment carries the risk of arterial thrombosis and leads to renal infarction. which causes abdominal or flank pain, that usually resolves after normalization of blood flow through the kidney.

It was difficult to determine the probable cause of the pseudoaneurysm in our patient because the aneurysm began to form gradually about a month after the procedure. The probable causes of a pseudoaneurysm include incorrectly or insufficiently applied pressure dressing, coagulation disorders, and too early or too intense physical activity (for example lifting weights) by the patient. This case highlights the importance of careful monitoring of access sites and the potential need for surgical intervention even after successful embolization.

## Conclusions

Renal AVM should be included as a potential alternative diagnosis for various symptoms, such as hematuria and hypertension resistant to medication. Although endovascular embolization is a less invasive, safer, and more effective treatment option in comparison with open surgery, it has a potential risk of complications, including renal infarction and pulmonary embolism. The success is dictated by complete occlusion of the shunted vessel while preventing the migration of embolic materials and preserving normal arterial branches. It depends on the selection of adequate techniques and embolic materials for individual cases, based on etiology and precise vascular architecture assessment.
